# Microencapsulation of Photochromic Solution with Polyurea by Interfacial Polymerization

**DOI:** 10.3390/polym13183049

**Published:** 2021-09-09

**Authors:** Yuhua Zhang, Xi Zhang, Yurong Yan, Zhonghua Chen

**Affiliations:** School of Material Science and Engineering, South China University of Technology, Guangzhou 510640, China; zhangyuhua_scut@hotmail.com (Y.Z.); zhxxi333@163.com (X.Z.)

**Keywords:** photochromic material, photochromic property, microcapsules, photostabilizer, interfacial polymerization, polyurea

## Abstract

Photochromic materials are interesting materials because of their color-changing property under UV light and visible light irradiation. However, they are vulnerable to many factors, such as pH oxygen, ion, solvent, etc. because of the unsaturated bonds existing on the photochromic molecular. Microencapsulation of the photochromic materials can separate them from the surroundings. Here, photochromic microcapsules using 3,3-Diphenyl-3H-naphtho[2,1-b] pyran (NP)/solution as core and polyurea as shell via interfacial polymerization were prepared, and bis(2,2,6,6-tetramethyl-4-piperidinyl)sebacate (HALS 770) was used as photostabilizer. Fourier transform infrared spectroscopy (FTIR), a laser particle size analyzer, a scanning electron microscope (SEM), a thermogravimetric analyzer and an ultraviolet-visible spectrophotometer were used for characterization. The results showed that the microcapsules had a uniform particle size of about 0.56 μm when the percentage of the oil phase (core) in the emulsion was less than 15%, the addition amount of the emulsifier was 0.4%, and the stirring rate was 1800 r/min. The microcapsules showed better performance in thermal stability when the core/shell ratio was 1:1. The photostabilizer had little impact on the color-changing property of the microcapsule, but it could protect the microcapsules from UV light radiation aging.

## 1. Introduction

Photochromic materials are compounds that can change color when exposed to UV light or visible light [1]. The characteristic of photochromic materials has attracted great attention among researchers in different fields, such as optical anti-counterfeiting [2,3], smart fiber [4], information storage [5], color-changing film [6], and color-changing glass [7,8]. To make better use of photochromic materials, researchers have made a lot of effort to synthesize or modify photochromic molecules. Vyasamudri et al. [9] prepared colorless 2,6-dioxabicycles and 2,8-dioxabicycles using the method of divergent synthesis. They found the 2,6-dioxabicycles synthesized by base catalysis could turn red when exposed to UV light. Helmy et al. [10] designed a new kind of photochromic molecule with properties of good fatigue resistance and color-switching under visible light. It is significant in many fields, for example, biosensors and targeted delivery systems. Researchers brought photochromic groups or molecules into the main chain or substituted side group of polymers via covalent bonds, and designed modified functional polymers characterized by photochromic properties [11,12,13,14,15].

Compared to inorganic photochromic material, organic photochromic materials respond quickly to light and can restore to their original state rapidly without light [16], which brings them more interest in use. However, unsaturated bonds typically present on the organic molecule of the photochromic materials which are sensitive to pH, oxygen, ion, solvent etc. leading to a shortened service life in actual application [17]. In addition, some organic photochromic materials show better photochromic properties only when dissolved in a specific solvent [18,19,20]. Thus, in order to use them practically, we should separate them from their surroundings and keep them in shape. Microencapsulation is an effective way to make this a reality. As a relatively mature technology, microencapsulation has been applied in many fields [21,22,23,24,25]. The most common ones are phase change material microencapsulation and drug sustained-release microencapsulation [26,27,28]. There is also some literature about microencapsulation of photochromic materials [29,30,31,32]. Feczkó et al. [29] prepared a kind of photochromic nanocapsule using poly (methyl methacrylate) and ethyl cellulose as shell and spirooxazine solution as core by the emulsion–solvent evaporation method. They found that the color changed more intensely and lasted longer compared to the solution of the same spirooxazine in an organic solvent. However, there exists a large amount of solvent evaporation in the preparation of the microcapsules, which is not environmentally friendly, so it needs further treatment of the solvent vapor. Zhou et al. [30] microencapsulated photochromic compounds with melamine-formaldehyde successfully and applied them to textile. The lifetime of photochromic compounds after encapsulation could extend 10 times compared with before encapsulation under continuous UV. However, melamine-formaldehyde will release formaldehyde when used, which is harmful to human health. Polyurea is a promising wall material in microcapsules due to its chemical stability, acid and alkali resistance, and solvent resistance. Usually, polyurea shell microcapsules were obtained via interfacial polymerization. The polyfunctional isocyanate was introduced to the oil phase and the polyfunctional amine was added to the water phase, then a condensation reaction occurred at the interface of the oil and water phases [33]. Polyurea has been used to encapsulate butyl stearate containing spirooxazine to form reversible photochromic energy storage microcapsules by Sun et al. [34].

Since most microcapsules are designed with polymers as shells, which decompose easily when exposed to UV light [35], the microcapsule of photochromic material may suffer aging and decomposition during their use. Moreover, the photochromic materials also suffer from photooxidation during their color-changing under UV light irradiation. Photostabilizer can quench the excited state of the molecule or group excited by UV rays and return it to the ground state, eliminating or slowing down the possibility of photo-redox reaction [36]. It is always used in polymer products to reduce the damage from UV light. However, to the best of our knowledge, the reports about photostabilizer used in microcapsules are rare. In this paper, we tried to microencapsulate a kind of photochromic compound with polyurea by simple interfacial polymerization. We creatively addicted bis(2,2,6,6-tetramethyl-4-piperidinyl)sebacate as photostabilizer to the microcapsules. Particle size distribution, morphology, thermo-stability, and the photochromic property of the microcapsules were investigated.

## 2. Materials and Methods

### 2.1. Materials

3,3-Diphenyl-3H-naphtho[2,1-b] pyran (NP) (purity >98%) purchased from Aladdin Reagent (Shanghai) Co., Ltd. (Shanghai, China) was used as photochromic material; S-1000 (a mixture of non-volatile hydrocarbons) supplied by Xinxiang Bailu Chemical Fiber Group Co., Ltd. (Xinxiang, China) was used as the solvent of photochromic material; isophorone diisocyanate (IPDI) (purity >99%) bought from Aladdin Reagent (Shanghai) Co., Ltd. and diethylenetriamine (DETA) (purity >98%) provided by Fortune (Tianjin) Chemical Reagent Co., Ltd. (Tianjin, China) were used as monomers of the polymer shell; bis(2,2,6,6-tetramethyl-4-piperidinyl)sebacate (HALS 770) (purity >99%) supplied by Aladdin Reagent (Shanghai) Co., Ltd. (Shanghai, China) was employed as photostabilizer; Tween 80 (Reagent Grade) purchased from Aladdin Reagent (Shanghai) Co., Ltd. (Shanghai, China) was used as emulsifier. All the materials were used without any further purification.

### 2.2. Synthesis of the Photochromic Material Microcapsule

**Figure 1:**
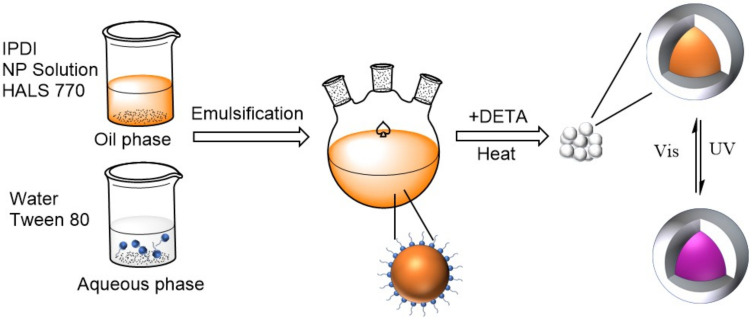
Scheme of the synthesis of the photochromic material microcapsule.

Microcapsules containing photochromic material were prepared by emulsion interfacial polymerization. Firstly, NP and HALS 770 were dissolved in the solvent at 50 °C to form the photochromic solution, after the solution was cooled to room temperature, IPDI was mixed with it as the oil phase; A certain amount of deionized water and Tween 80 were added to the three-neck flask and stirred mechanically using a high shear mixer to form the aqueous phase; The oil phase was dropped to the aqueous phase slowly under the vigorous stirring of the shear mixer at room temperature; After the completion of the oil phase dripping, it was stirred for 30 min and then the required emulsion was achieved. The stirring rate was set at 600 r/min and 10% (*w*/*w*) aqueous solution of DETA was added to the emulsion drop by drop using a constant pressure funnel. The reaction lasted for 4 h at room temperature and continued for 8 h at 50 °C. The suspension of microcapsules was achieved after the final 2 h at 80 °C. Finally, the production was washed by petroleum ether three times and then dried in the fume hood. The microencapsulation process is schematically shown in Figure 1.

### 2.3. Characterization

#### 2.3.1. Fourier Transform Infrared Spectroscopy (FTIR)

The chemical structure of 3,3-diphenyl-3H-naphtho[2,1-b]pyran (NP) solution and its microcapsule were characterized by FTIR. The spectra of photochromic solution were collected by dropping the liquid on the potassium bromide (KBr) pellets directly, and the spectra of microcapsules were collected by mixing the solid with KBr pellets. All spectra were obtained via 32 scans from 4000 to 400 cm^−1^ using an FTIR spectrometer (Bruker Dalton, Tensor II).

#### 2.3.2. Particle Size and Its Distribution

The particle size and the distribution of the microcapsules were investigated using a laser particle size analyzer (Beckman Coulter, LS13320). The tested microcapsule was dispersed in deionized water via ultrasonic dispersion by the cell disruptor (Lichen, LC-JY88-IIN). Then the particle size and its distribution were measured by dropping the dispersions to the sample chamber of the laser particle size analyzer.

#### 2.3.3. Surface Features and Morphology

The surface features and morphology of the microcapsules were observed on a scanning electron microscope (SEM, Holland, NOVA NANOSEM 430). A sprinkle of sample was put on electric conductive adhesive and coated with a layer of platinum to avoid being scorched.

#### 2.3.4. Thermostability

The thermal decomposition temperatures of the microcapsules were analyzed using a thermogravimetric analyzer (TGA, Netzsch, HAAKE400P). We placed 5–10 mg sample of the microcapsule in an alumina crucible and heated it at the rate of 10 °C/min from 30 to 600 °C under N_2_ atmosphere. The weight of the tested sample lost in different ranges and the temperature points at which the sample started to lose weight were recorded on the curves of TGA.

#### 2.3.5. Tightness Performance

The tightness performance of the microcapsules was tested by observing the weight loss of the microcapsules. 10 g sample of the product was placed in an oven at 80 °C for 24 h, and the percentages of the remaining mass were recorded every four hours.

#### 2.3.6. Ultraviolet–Visible (UV-Vis) Absorbance

The UV-Vis spectra of the microcapsules were performed by an ultraviolet-visible spectrophotometer (Hitachi, Japan, U-3010).

#### 2.3.7. Photochromic Cycle Test

The suspensions of the microcapsules were put into a colorless transparent glass screw bottle, then the samples were irradiated by UV light with wavelength of 365 nm using a flashlight (5 W, Ultra fire, 501 B) for 2 min and kept in the dark for 10 min alternately.

#### 2.3.8. UV Accelerated Aging Test

Microcapsule suspensions were exposed to the UV light (40 W/m^2^, 340 nm) at a distance of 30 cm in an ultraviolet spectrum accelerated aging test machine (Q-Lab, QUV/Spray) at 25 ± 5 °C for 15 d [37]. After that, the absorbance at the strongest absorption wavelength (550 nm) of the microcapsules was measured via a UV-visible spectrophotometer (Hitachi, Japan, U-3010).

#### 2.3.9. Characterization of Color Change

The color change of the microcapsules was tested by recording the chromatic aberration(ΔE) of the microcapsules before and after the irradiation of the UV light.

## 3. Results and Discussion

### 3.1. Preparation of the Photochromic Microcapsule

The photochromic microcapsules were prepared via interfacial polymerization of IPDI and DETA in the emulsion. At first, IPDI in the oil phase was dispersed into the water uniformly to form an O/W emulsion. Subsequently, DETA solution was dropped into the O/W emulsion slowly. The –NCO group can react easily with lots of molecular groups having active hydrogen [38], for example, –OH, –NH_2_, –NH–, –COOH. But the reaction rate varies widely. The reactivity of –NH_2_ is much higher than other groups. Therefore, the –NH_2_ groups on DETA reacted with –NCO groups on IPDI immediately at the interface of oil phase and aqueous phase once the DETA solution was added to the emulsion. In order to control the reaction rate of IPDI with DETA, it is significant to slow the speed of DETA’s addition. When finishing the addition of DETA, the concentrations of –NCO and –NH_2_ decreased as the reaction lasted. It was necessary to raise the reaction temperature to 50 °C to ensure the continued reaction of IPDI with DETA. The shell formed gradually at the surface of the oil phase dispersion as the polymer chains’ length increased. In the final stage of the reaction, –NH– on the polymer chains formed reacted with –NCO groups on IPDI at a higher temperature of 80 °C, which caused the cross-linking of the polymers. In this process, IPDI might also react with water theoretically because of the –OH existing on the water molecule. However, the reactivity of –OH group on water is much lower than that of –NH_2_ and –NH, so the reaction mainly occurred between IPDI and DETA.

### 3.2. FTIR

To chemically confirm the component of the microcapsule of the photochromic solution, FTIR Spectrometer was employed to achieve the spectra of photochromic solution, blank polyurea of IPDI and DETA, as well as microcapsules.

**Figure 2:**
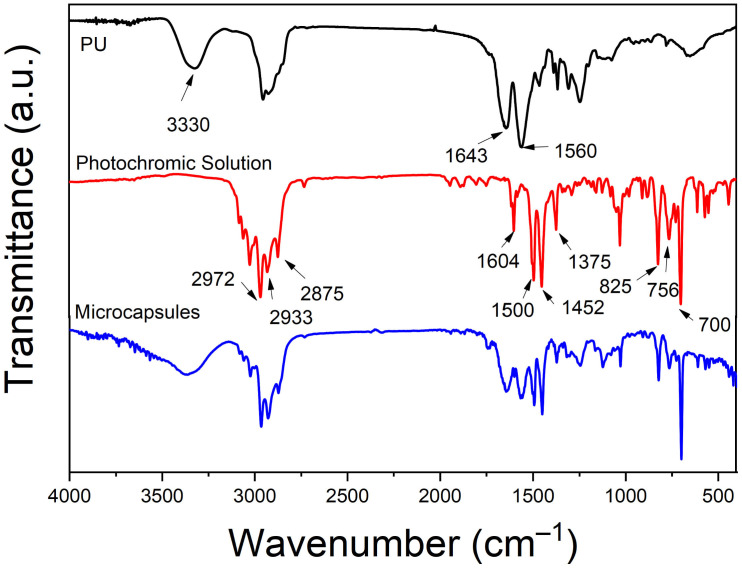
The infrared spectrum of NP solution, PU, and microcapsules.

The FTIR spectra of photochromic solution, polyurea, and microcapsules are shown in Figure 2. In the spectrum of the photochromic solution, the bands at 2875 cm^−1^ and 2972 cm^−1^ could belong to the symmetric and asymmetric stretching vibration of –CH_3_, respectively. The peaks of 1375 cm^−1^ and 1452 cm^−1^ may be attributed to the symmetric and asymmetric formation vibration of –CH_3_ respectively, which also illustrate the existence of –CH_3_. The peak at 2933 cm^−1^ is the stretching vibration of –CH_2_. The benzene ring is certified to exist by a series of peaks at 1604, 1500 cm^−1^, which belong to the vibration of benzene ring carbon backbone, and 825, 756, 700 cm^−1^, which are attributed to the bending vibration of –CH on benzene. In the spectrum of polyurea, a strong stretching vibration of –C=O appears at the peak of 1643 cm^−1^; The bending vibration of the –NH peak is observed at 1560 cm^−1^, and a broad peak around 3350–3200 cm^−1^ is the stretching vibration of −NH. Besides, there does not exist the peak around 2260 cm^−1^, which is to indicate the existence of –NCO, so it demonstrates that the IPDI monomers had fully reacted. All peaks on the aforementioned spectra appear on the spectrum of the microcapsule and there are no other new peaks appearing; therefore, we can conclude that the microcapsule is composed of the photochromic solution and polyurea, and the monomers do not react with the photochromic solution.

### 3.3. Particle Size and Its Distribution

The particle size and distribution of microcapsules are affected by many factors in the preparation. Among them, the initial addition amount of raw materials, emulsifier, and stirring rate of emulsification are worth noting.

**Figure 3:**
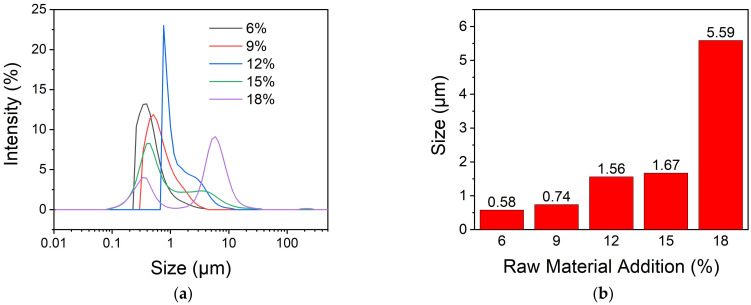
The size and size distribution of microcapsules prepared with different addition amount of oil phase (**a**) and the average diameters (**b**).

The particle size of microcapsules prepared with different amounts of oil phase solution was measured. The result was shown in Figure 3. From Figure 3, it can be seen that the microcapsules have relatively narrow size distributions with average diameters of 0.58 and 0.74 μm when the percentage of the oil phase in the emulsion is 6% and 9%, respectively. With the addition amount increasing, the size distribution becomes broader. When the percentage of the oil phase is larger than 15%, two peaks appear on the curve of the size distribution of microcapsules and the average diameters become larger. This may be due to a high concentration of dispersed phase. As the concentration of the dispersed phase in the emulsion increases, the probability of collisions between dispersed phase particles increases, so the coalescence becomes easier for the emulsion. Moreover, the reaction rate becomes larger because the concentration of IPDI increases with the dispersed phase, which leads to the agglomeration of microcapsules. Therefore, for the purpose of obtaining uniform microcapsules, it is better to add raw materials of not more than 15% in weight.

**Figure 4:**
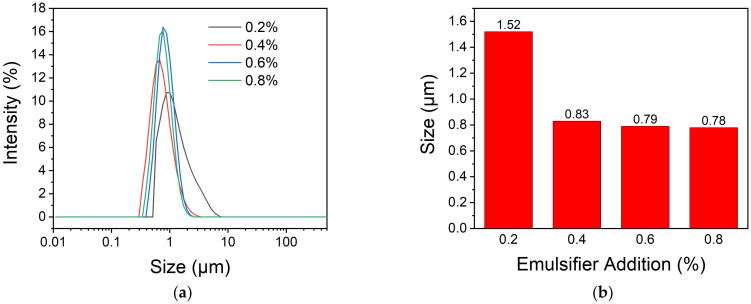
The size and size distribution of microcapsules prepared with different addition amount of emulsifier (**a**) and the average diameters (**b**).

Emulsifier plays an important role in decreasing the interfacial tension and minimizing the effects of interfacial forces in emulsions. Figure 4 shows the influence of emulsifier addition on the particle size and particle size distribution. It can be seen that the particle size distributions become narrow and the average diameters decrease with the increasing addition of the emulsifier. The microcapsules’ average diameter reduces to 0.83 μm from 1.52 μm as the addition amount of emulsifier increases to 0.4% from 0.2%. With a further increase dosage of emulsifier, the particle size decreases slightly. As we know, the emulsion forms relying on the emulsifier surrounding the surface of dispersed droplets. The smaller the droplets are, the larger the area of the interface formed in the emulsion, and the more emulsifier the molecules need. There are not enough emulsifier molecules to form a stable layer around the emulsion droplets when the emulsifier addition is 0.2%, so the droplets tend to agglomerate to form larger droplets. When the percentage of emulsifier exceeds 0.4%, the emulsifier is not the main factor to affect the formation of emulsion anymore, therefore the microcapsules’ size does not decrease that obviously with more emulsifier added. As a consequence, microcapsules with relatively small diameter and narrow particle distribution were prepared with an emulsifier addition of 0.4%.

**Figure 5:**
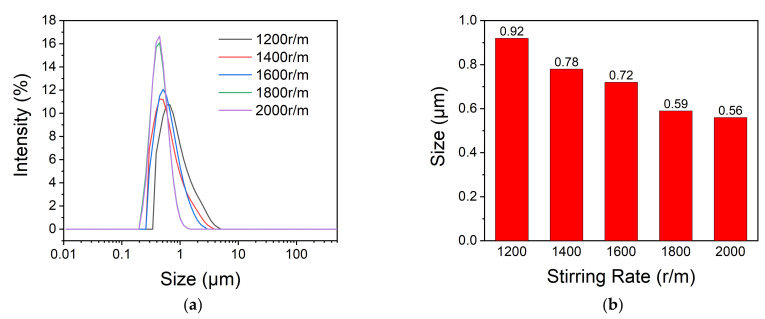
The size and size distribution of microcapsules prepared under different stirring rates (**a**) and the average diameters (**b**).

The shearing force of core material is obtained through mechanical stirring. Different stirring rates provide different shearing forces for the emulsion, hence, the stirring rate affects the formation of the emulsion. As shown in Figure 5, the size of the microcapsules becomes small and the particle size distribution becomes narrow gradually as the stirring rate becomes large. The average diameter of the microcapsules decreases from 0.92 μm to 0.56 μm when the stirring rate increase from 1200 r/min to 2000 r/min. This may be explained as follows: emulsions are not thermodynamically stable, and the dispersed droplets will agglomerate and adhere together when without external force. Mechanical stirring can put a shearing force to the oil phase and there is a positive correlation between shearing force with the stirring rate. When the stirring rate increases, the shearing force becomes large, which leads to the droplets keeping a balance of dispersing and agglomeration in a smaller size. However, the diameters of microcapsules prepared at the stirring rate of 1800 r/min and 2000 r/min are not much of a distinction, so the stirring rate of 1800 r/min is good enough for the emulsion system.

### 3.4. Morphology of the Microcapsules

**Figure 6:**
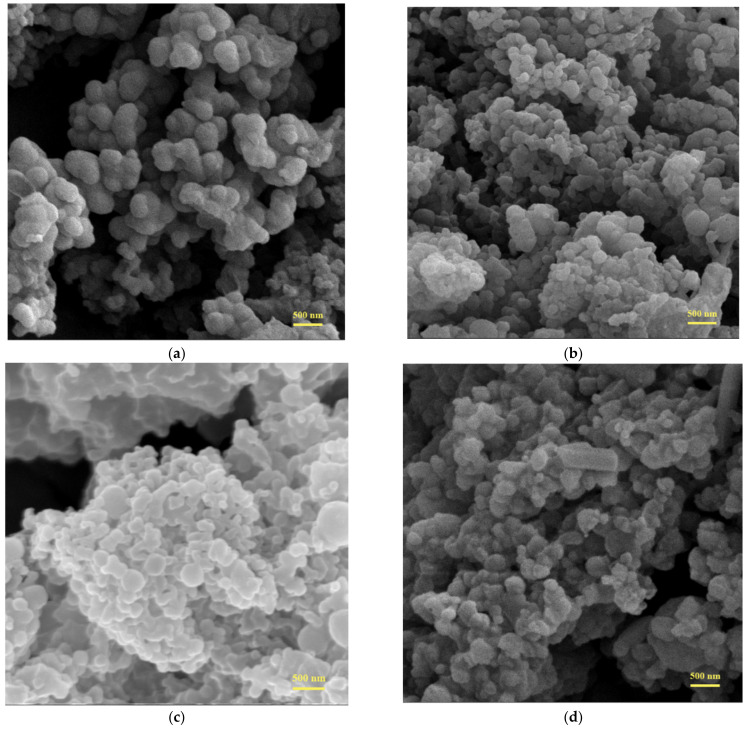
SEM of the microcapsules prepared by different core/shell ratio: (**a**) 1:1 (**b**) 2:1 (**c**) 3:1 (**d**) 4:1.

The morphology of the microcapsules prepared with different ratios of core material and shell material was evaluated via SEM. The SEM images of microcapsules are shown in Figure 6. It can be seen that all the microcapsules are almost spherical. The diameters observed on the SEM images range from 0.2 to 0.4 μm, which is slightly smaller than that measured by the laser particle size analyzer. There may be two reasons for this. On one hand, we obtained the particle size using the laser particle size analyzer by recording the Brownian motion of particles in the dispersion, so there is a certain deviation from the value obtained by SEM. On the other hand, the microcapsules agglomerate together easily, it is hard to obtain the suspension with single microcapsules dispersed. Therefore, the diameters we measured via the laser particle size analyzer were actually the agglomerated microcapsules.

From Figure 6, we can see that the microcapsules could be formed with different dosages of shell material. However, there are obvious differences in shape and morphology of the microcapsules of different core/shell ratios. From Figure 6c,d, it can be seen that the microcapsules synthesized with core/shell ratios of 4:1 and 3:1 cluster more seriously compared with those synthesized with core/shell ratios of 2:1 and 1:1. Moreover, there appear crystals when the core/shell ratio is 4:1, which illustrates that the encapsulation performance of the microcapsules is too poor so the core materials will seep out gradually. From Figure 6a,b, we can observe that the microcapsules are more uniform and the surfaces are more smooth when the core/shell ratio is 1:1. In summary, better microcapsules were obtained with the increased amount of shell material, so in order to guarantee the encapsulation property of the microcapsules, we should appropriately increase the shell material when preparing the microcapsules. In this work, the core/shell ratio of 1:1 is relatively suitable for the formation of the microcapsules.

### 3.5. Thermogravimetric Analysis (TGA)

**Figure 7:**
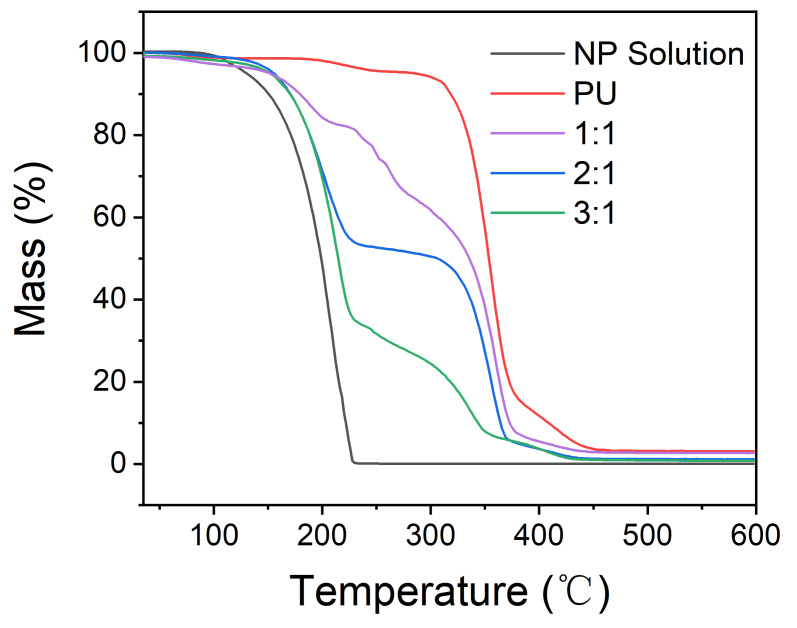
TGA curves of PU, NP solution, and microcapsules with core/shell ratio of 1:1, 2:1, and 3:1.

To investigate the thermostability of the microcapsules and to calculate the core content encapsulated in the microcapsules, TGA curves of the microcapsules prepared with different ratios of core material and shell material were obtained via a thermogravimetric analyzer. As discussed in the analysis of SEM, the microcapsules are not that reliable when the core/shell ratio is 4:1, so we ignore the thermogravimetric analysis of them prepared with the core/shell ratio of 4:1. Figure 7 is the TGA results of shell material, photochromic solution, and microcapsules prepared with different core/shell ratios, and the details of the decomposition temperatures and char yield of the samples are shown in Table 1. According to the TGA curves, the photochromic solution began to lose weight at 149 °C (T_10%_), which was mainly caused by the evaporation of the solvent. The shell material of polyurea (PU) synthesize by IPDI and DETA is quite stable to temperature. The PU shell without core solution started to break down until the temperature rises to 317 °C and decomposed completely at about 465 °C, which led to a sharp drop of the TGA curve of PU at 317–465 °C [33]. Considering the microcapsules fabricated with core/shell ratios of 1:1, 2:1, and 3:1, there were two stages of weight loss in the process of temperature rise on all TGA curves of microcapsules. The first began at about 169 to 178 °C and the second began at about 317 °C; they were attributed to the evaporation of the core solution and the decomposition of the shell, respectively. The temperature at which photochromic solution in microcapsules starts to lose weight is higher than that without encapsulation. This proves that the microcapsules can protect the photochromic solution from evaporation to a certain degree. The decomposition temperature of the microcapsules’ shell is in correspondence with that of PU synthesized by IPDI and DETA, which illustrates the shell material keeps its performance when used in microcapsules. It is worth noting that there exists an obvious difference on the TGA curves of microcapsules with core/shell ratios of 1:1 compared to that of 2:1 and 3:1. Both the microcapsules with core/shell ratios of 2:1 and 3:1 lost weight rapidly at the temperature from 169 to 240 °C and then had a platform until the shell material started to break down, However, when the microcapsules’ core/shell ratio was 1:1, the TGA curve dropped nearly linearly with a slow rate at the temperature from 178 to 308 °C. Microcapsules could prevent the formed vapor from volatilizing because the thick shell formed when the core/shell ratio was 1:1. When the core/shell ratios were 2:1 and 3:1, the shell of the formed microcapsules might be too thin to keep the core solution from permeating out at a relatively high temperature. Therefore, to make the microcapsules better in encapsulation performance, increasing the shell material amount is necessary.

**Figure 8:**
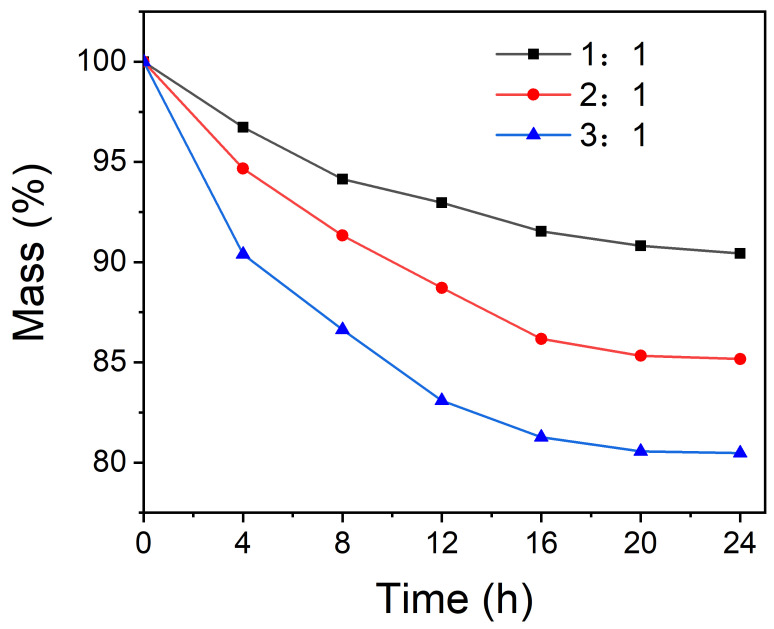
The mass loss of the microcapsules with core/shell ratio of 1:1, 2:1 and 3:1 at 80 °C.

To further confirm the microcapsules’ stability of encapsulation, the samples were heated to 80 °C in an oven, and the masses were recorded every 4 h. The result was shown in Figure 8. We can conclude that with the increase of core/shell ratio, the tightness of the microcapsules becomes worse, so the mess loss increased. This is consistent with the result of the TGA analysis.

### 3.6. Photochromic Performance

**Figure 9:**
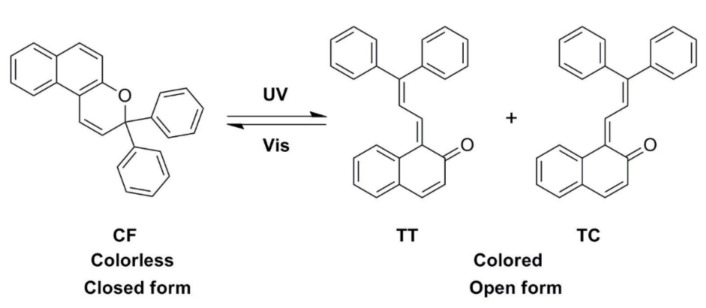
Reversible structural transformation of 3,3-diphenyl-3H-naphtho[2,1-b] pyran (NP) under UV and visible light.

**Figure 10:**
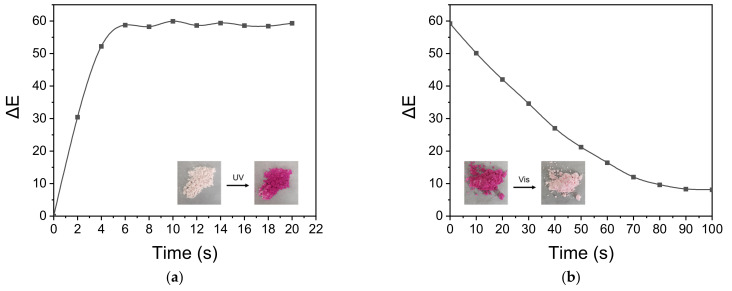
The chromatic aberration (ΔE) of the microcapsules under UV irradiation (**a**) and the fading time under visible light (**b**).

As seen in Figure 9, reversible structural transformation occurs in 3,3-Diphenyl-3H-naphtho[2,1-b] pyran (NP) under UV and visible light [39]. The colorless form (CF) undergoes a ring-opening reaction to form two kinds of colored forms of cis (TC) and trans (TT) structures under the UV light. On the contrary, the colored forms revert to the original structure when the UV light is removed. The responding time of the microcapsules’ color-changing was tested by recording the chromatic aberration (ΔE). The result was shown in Figure 10. We can see that the chromatic aberration of the microcapsules reached the maximum value after 6 s UV light irradiation. After that, when we continued to extend the time of UV light irradiation to the samples, the chromatic aberration was almost unchanged, which illustrated the reaction reaches a state of dynamic equilibrium. When the UV light was removed, the chromatic aberration decreased gradually and it took about 90 s to restore to the original color, which meant the colored form of the TF and TT structures reverted to the colorless form of CF.

As we know, polymers break down easily under the irradiation of UV light. It is meaningful to add the photostabilizer to the polymers to reduce the impact of the UV light [40,41]. In the photochromic microcapsules in particular, UV light is necessary to obtain color-changing. In order to extend the service life of the microcapsules, we applied the bis(2,2,6,6-tetramethyl-4-piperidinyl)sebacate (HALS 770) as the photostabilizer to the microcapsules.

**Figure 11:**
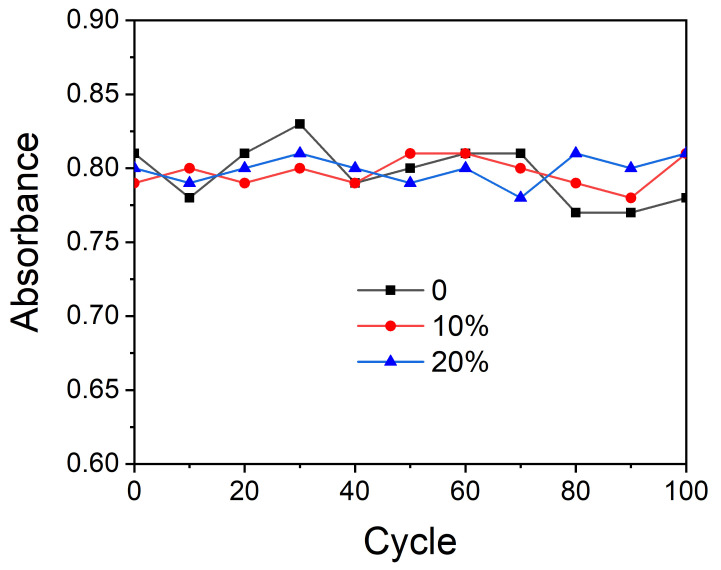
The effect of HALS 770 on the absorbance of the microcapsules.

For the purpose of investigating the effect of photostabilizer on the color-changing of the photochromic microcapsules, samples with different amounts of HALS 770 were obtained. We tested the absorbance of the microcapsules suspension at 550 nm after UV light irradiation. Figure 11 showed the result of 100 color-changing cycles. We can see that the absorbances of the microcapsules with different amounts of HALS 770 were nearly constant, which meant the addition of the photostabilizer had little impact on the absorbance of the microcapsules. Both the microcapsules with and without photostabilizer have excellent fatigue resistance. Table 2 showed the chromatic aberration of microcapsules with different amounts of HALS 770 before and after UV light irradiation after 100 color-changing cycles. The results illustrate that all the samples kept a good color-changing property, and the chromatic aberration values remained above 30, which meant there was a significant color change before and after UV light irradiation to the microcapsules.

**Figure 12:**
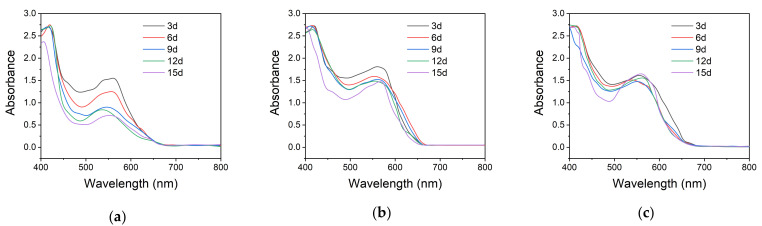
The absorbance of the microcapsules with different content of HALS 770 during the UV accelerated aging test. The content of HALS 770 in the microcapsules: (**a**) 0; (**b**) 10%; (**c**) 20%.

To further corroborate the effect of the photostabilizer on the photochromic microcapsules, a UV accelerated aging test was carried out. The absorbances of the microcapsules with 0, 10% and 20% (compared to the amount of NP) addition of HALS 770 were shown in Figure 12. According to Figure 12a, when the addition of the photostabilizer was 0, the absorbance of the microcapsules at 550 nm decreased rapidly as the UV accelerated aging test time increased, and the absorbance at 550 nm was less than a half after 15 d. From Figure 12b, when the addition amount of the photostabilizer was 10%, the absorbance of the microcapsules at 550 nm decreased slightly in the first 6 d, and then it remained nearly unchanged in the following UV accelerated aging test. As shown in Figure 12c, when the addition amount of HALS 770 increased to 20%, the absorbance of the microcapsules hardly declined at the UV accelerated aging test. We can conclude that the addition of HALS 770 can protect the microcapsules from the damage of the UV light so it can extend the service life of the microcapsules. The mechanism for the stabilization of microcapsules may be explained as follows [42]: free alkoxyl radicals and free alkyl radicals are formed when the microcapsules are exposed to UV light. HALS 770 possesses alicyclic amines that can be transformed into regenerating free nitroxyl radicals. The free nitroxyl can catch free alkoxyl and alkyl radicals formed during the aging of the microcapsules to form the appropriate esters. The esters formed can react with free radicals to regenerate free nitroxyl radicals and build organic molecular chains, so a large number of free radicals is consumed, resulting in the delay of the aging process. When the addition of HALS 770 was 10%, the concentration of HALS 770 might not be high enough to consume the free radicals formed during the aging test, so the microcapsules decompose partially, leading to the decrease of the absorbance of the microcapsules at 550 nm. When the addition of HALS 770 reached 20%, the free radicals could be consumed relatively completely, so the microcapsules had a good performance in the aging test.

## 4. Conclusions

In this work, photochromic microcapsules had been prepared via interfacial polymerization. Polyurea synthesized by IPDI and DETA was suitable for the shell material in the microcapsules. The FTIR spectra demonstrated that the microcapsules consisted of the photochromic solution and polyurea. The percentage of the oil phase in the emulsion, addition of emulsifier and stirring rate had a great effect on the particle size and particle size distribution. When the percentage of the oil phase was less than 15% and the dosage of the emulsifier was 0.4% and the stirring rate was 1800 r/min, the microcapsules had a relatively uniform particle size of about 0.56 μm. The core/shell ratio impacted the micromorphology and the stability of the microcapsules. When the core/shell ratio was 1:1, the microcapsules had good performance in thermal stability. After encapsulation, the thermal weight loss temperature increased from 149 to 178 °C. The microcapsules had a very fast response rate to the UV light, and it took only 6 s to reach the max chromatic aberration value for the microcapsules under the UV light irradiation. HALS 770 as the photostabilizer had little impact on the color-changing property. The microcapsules with and without HALS 770 showed an excellent performance on the color-changing cycle property. However, HALS 770 could affect the UV aging resistance of the microcapsules. When adding 20% HALS 770, the absorbance of microcapsules kept nearly consistent during the UV accelerated aging test, which meant HALS 770 could protect the microcapsule from the UV light damage. This research provides new insight into the application of photochromic materials, and there are some other issues worthy of further study in the follow-up work, such as the thickness of the microcapsules’ shell, the method to reduce agglomeration of microcapsules, and the influence of solvent on the microcapsules’ photochromic property, etc.

## Figures and Tables

**Table 1:** Results of the thermogravimetric analysis of PU, NP solution, and microcapsules with core/shell ratio of 1:1, 2:1 and 3:1.

Samples	T_10%_ (°C)	Char Yield (%)
NP Solution	149	0.04
PU	317	3.10
Core/shell ratio of 1:1	178	2.69
Core/shell ratio of 2:1	171	1.12
Core/shell ratio of 3:1	169	0.79

Note: The decomposition temperatures of the samples we used were the temperatures at which the samples’ thermal weight losses were 10%, marked as T_10%_.

**Table 2:** The color parameter of microcapsules with different content of HALS 770.

		Content of HALS 770 (%)		
		0	10	20
Before UV light irradiation	L	84	84	82
	a	6	7	7
	b	6	6	6
After UV light irradiation	L	67	71	73
	a	33	32	34
	b	−12	−9	−16
ΔE		36.63	31.92	35.97

## Data Availability

The data presented in this study are available on request from the corresponding author.
